# Analysis of Rejuvenating Fiber Asphalt Mixtures’ Performance and Economic Aspects in High-Temperature Moisture Susceptibility

**DOI:** 10.3390/ma15217728

**Published:** 2022-11-02

**Authors:** Yao Zhang, Ye Wang, Aihong Kang, Zhengguang Wu, Bo Li, Chen Zhang, Zhe Wu

**Affiliations:** 1 Department of Transportation Engineering, College of Architectural Science and Engineering, Yangzhou University, Yangzhou 225127, China; 2 Jiangsu Aoxin Science & Technology Company, Yangzhou 225800, China

**Keywords:** hot rejuvenated asphalt mixtures, Hamburg wheel tracking test, moisture damage, economic benefit, fiber

## Abstract

Non-renewable resources such as natural stone and asphalt are in short supply. Recycling technology, with its lower cost, has been used as the primary approach to asphalt pavement maintenance engineering. The inclusion of reclaimed asphalt pavement materials in producing new asphalt pavements may increase the risk of cracking. The strength and toughness of the asphalt mixture can be reduced. In this study, Hamburg wheel tracking tests (HWTT) were performed on rejuvenated asphalt mixtures with distinct maintenance processes. Different kinds of fibers have been used as additives to reinforce the rejuvenated asphalt mixtures. The HWTT rutting curve was identified as having three stages, including the post-compaction stage, the creep stage, and the stripping stage. The three-stage rutting curve model was used to determine the intersection point between the creep stage and stripping stage. The other two feature points (i.e., the post-compaction point and the stripping inflection point) were redefined with a new calculation method. Then, the rutting effect and stripping effect were separated with these feature points. The performance and economic benefits of fiber-reinforced rejuvenated asphalt mixtures were investigated through grey correlation analysis under the three maintenance processes. The feature points of the HWTT curve and the cost of the corresponding maintenance process were selected as the impact factors. Finally, the optimal scheme was developed by analyzing the influence of each factor on both performance and economic benefits.

## 1. Introduction

The use of recycled materials such as reclaimed asphalt pavement (RAP) in hot mix asphalt (HMA) has increased due to the rising cost of petroleum-based products and the negative environmental impact of carbon emissions associated with asphalt binder production [1]. However, the main challenge in incorporating the RAP into the asphalt mixture is the aged binder, which makes the mixture more susceptible to cracking [2,3,4]. As a result, the aged binder in the RAP tends to limit the high content of recycled material in the mixture. The need to reverse the negative impact of recycled materials in the HMA has prompted researchers to identify and implement innovative and feasible approaches.

Nahar et al. detected the fusion interface between the old and new asphalt by using atomic force microscopy (AFM) [5]. It was found that the old and new asphalt were not entirely miscible. The RAP was portrayed more like a “black stone”, which was directly encapsulated by the new asphalt. Therefore, it is unable to truly address the performance degradation brought on by the old asphalt. There are weak links in the recycled asphalt mixture. Zhou et al. proposed that the performance metrics of the rutting and fatigue cracking resistance of the SBS-RAP blended binders were opposed to each other [6]. New experimental methods need to be introduced to equilibrate two performances to choose the optimal RAP content. Cheng P et al. found that the freeze–thaw splitting strength ratio of hot rejuvenated asphalt mixtures (HRAM) presented a decreasing trend with the increase in RAP content [7]. The decreasing rate was slow when the RAP content was within the range of 15% to 40%, while the decreasing rate became faster beyond this range. Since the aromatic phenol and saturated hydrocarbons in the aged binder transformed into resins and asphaltenes, various rejuvenators have been developed to restore the ratio of aromatic phenol and saturated hydrocarbons in rejuvenated asphalt binder [8,9]. The rejuvenators can reverse the aging process and restore part of the binder properties [10,11]. However, the properties of the cracking resistance and moisture susceptibility of the HRAM are still poor.

The Hamburg wheel tracking test (HWTT) was introduced for the evaluation of cracking resistance and moisture susceptibility with a more accurate reflection of the properties of asphalt mixtures [12]. The test involved the real-time monitoring of the correlation between the number of load cycles and the rutting depth (RD). Test curves were divided into three main phases: (a) the post-compaction phase, (b) the creep phase, and (c) the stripping phase. The stripping inflection point (SIP) was determined by counting the number of load cycles on the HWTT curve at which a sharp increase in rut depth occurs [13]. The SIP was represented at the intersection of the fitted lines that characterize the creep phase and the stripping phase. However, different fitting point selections could lead to an inaccurate finding of SIP. This may cause a misjudgment of the cracking resistance and moisture susceptibility of asphalt mixtures. In investigations on the rutting of hot recycled asphalt mixtures in the HWTT, researchers have found that the rutting depth increased more quickly in the third stage but developed more slowly in the first two stages when increasing the RAP. The findings give an indication that the properties of the cracking resistance and moisture susceptibility of hot recycled asphalt mixtures need to be enhanced, especially at increasing recycled binder ratios and RAP contents [14].

Fiber is a kind of natural or synthetic material, which acts as a reinforcement of strength and stability in the HMA. The fiber network structure enhances the binder properties of the asphalt mixtures [15]. It can withstand a portion of the force, preventing the cracking of the asphalt mixtures [16]. The moisture susceptibility can be also improved with the addition of fibers, and different types of fibers have varied functions in asphalt mixtures [17,18]. Chen Y Z et al. found that fibers could significantly improve the residual stability and freeze–thaw splitting strength of asphalt mixtures [19]. In comparison to polyester and wood algal fibers, basalt fibers are more effective at increasing the water stability of asphalt mixtures. Zhang K et al. found that asphalt mixture containing basalt fiber achieved better results in resisting salinity and moisture environments [20]. Although adding fibers and recycling agents as well as new aggregates can perform good results in asphalt mixtures, doing so will certainly drive up the price of the HRAM. On the other side, the excessive use of RAP can reduce the cost of the HRAM, but it degrades the serviceability of asphalt mixtures.

**Figure 1:**
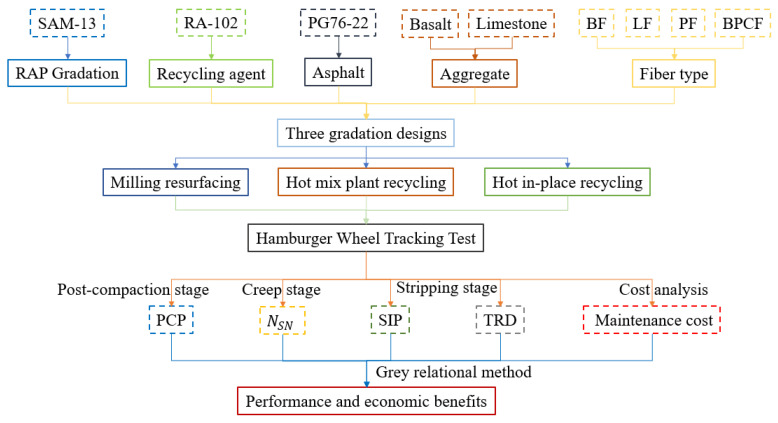
Flowchart of the research.

Hence, how to select a hot rejuvenated asphalt mixture that is both affordable and high-performing among available possibilities has been the main motivation for study. This is also the actual need of the pavement maintenance industry. Meanwhile, more innovative methods are needed to gain the feature points of the HWTT curve. Moreover, the properties of the cracking resistance and moisture susceptibility of hot recycled asphalt mixtures can be improved by controlling these feature points. Therefore, the objective of this paper is to investigate the mix design of hot rejuvenated asphalt mixtures with different fiber types under three pavement maintenance processes. The analysis approach for obtaining the feature points under three stages is modified after conducting the HWTT to obtain rutting curves. Subsequently, the costs of asphalt mixtures are obtained through an economic benefit analysis. Finally, the performance and economic benefits evaluation is conducted using grey correlation analysis to provide a rational solution and guidance for asphalt pavement maintenance projects. The flowchart of this study is shown below (Figure 1).

## 2. Construction Technology and Raw Materials

Three hot regeneration maintenance techniques were employed to create hot rejuvenated asphalt mixtures to investigate the high-temperature performance and moisture susceptibility of each asphalt mixture under various pavement maintenance operations in the HWTT.

### 2.1. Construction Technology

**Figure 2:**
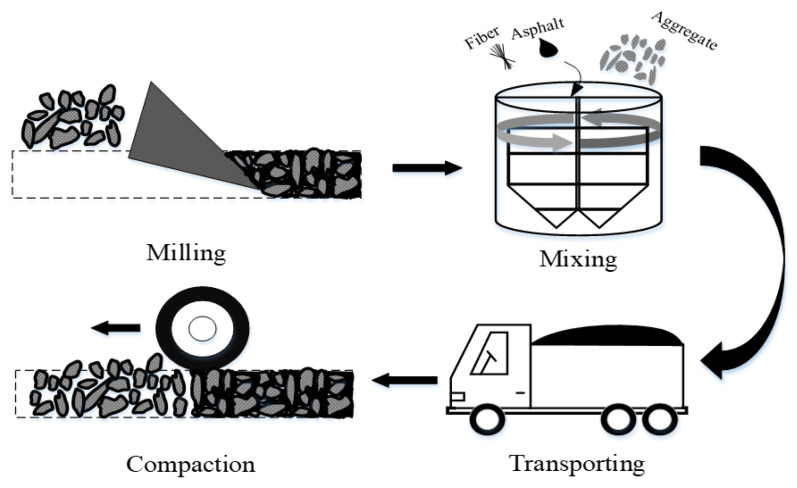
Milling resurfacing process.

**Figure 3:**
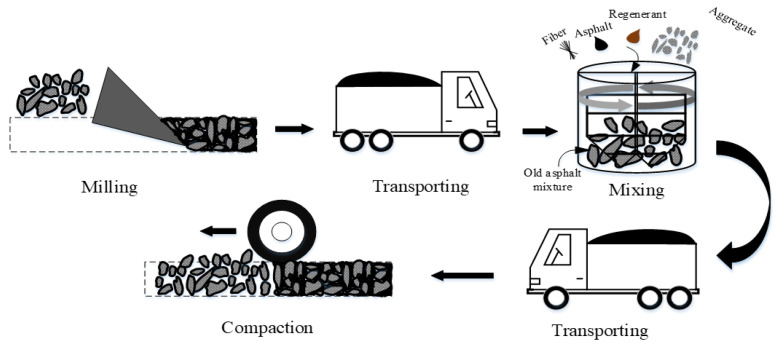
Hot mix plant recycling process.

**Figure 4:**
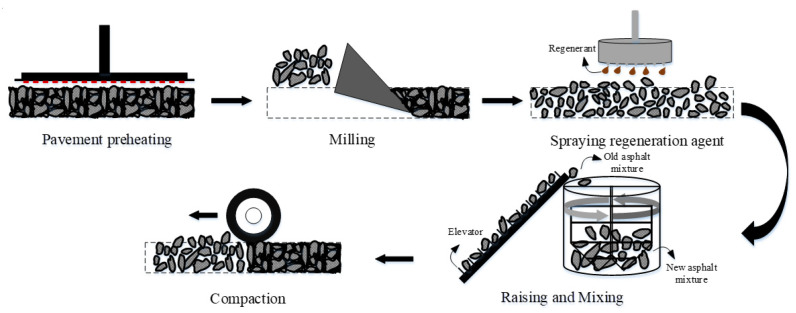
Hot in-place recycling process.

There are three typical maintenance technologies in current pavement construction, including the milling resurfacing (MR) process, the hot mix plant recycling (HMPR) process, and the hot in-place recycling (HIPR) process. The RAP dosage in the asphalt mixture differs greatly for the three maintenance processes. The RAP dosage in HIPR is generally more than 70%, while it is generally less than 50% in the HMPR process. There is no RAP used in the MR process; in other words, the material is a 100% new asphalt mixture. The basic principles of the three pavement maintenance processes are shown in Figure 2, Figure 3 and Figure 4.

In the MR process, the old asphalt pavement is milled, and then 100% of the new asphalt mixture from the asphalt mixing plant is spread and compacted on the milled pavement. During the HMPR process, the RAP is transported to the asphalt mixing plant as aggregates, where it is mixed with the new asphalt mixture after adding some of the recycling agents. In the HIPR process, 10% to 30% of the new materials are transported to the construction site. The old pavement is loosened by hot raking with the HIPR equipment. Then, the recycling agent and new asphalt mixtures are stirred with the old asphalt mixtures to create rejuvenated asphalt mixtures. Finally, the rejuvenated asphalt mixtures are paved in situ and compacted to form new pavement.

By comparison of the three maintenance technologies, it can be seen that the milling resurfacing process uses the highest quality raw materials to ensure excellent pavement performance. However, the cost of this process is rather high, and the old materials cannot be reclaimed, which can pollute the environment. The hot mix plant recycling process can solve part of the RAP recycling problem, and more than 70% of the raw materials are added in this process, which can also ensure the performance of asphalt pavements. The hot in-place recycling process allows for the complete use of old materials, significantly reducing material costs. It can also increase the efficiency of construction and reduce traffic. However, the incorporation of excessive RAP materials may affect the crack resistance of asphalt pavements. As seen from these pavement maintenance technologies, it is found that each of them has its characteristics. When choosing the appropriate technology for pavement maintenance, the construction process should take both the technical and financial benefits into account.

### 2.2. Raw Materials

#### 2.2.1. RAP

**Figure 5:**
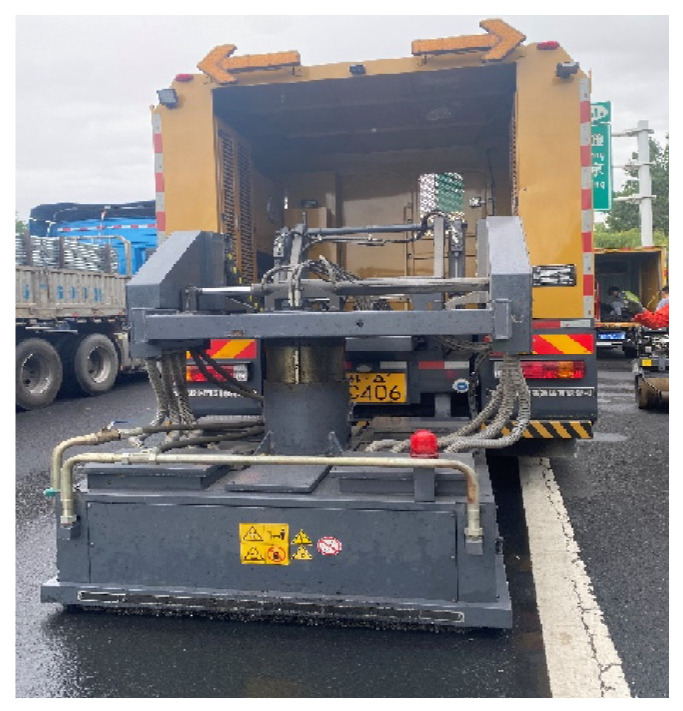
Pavement heating car.

**Figure 6:**
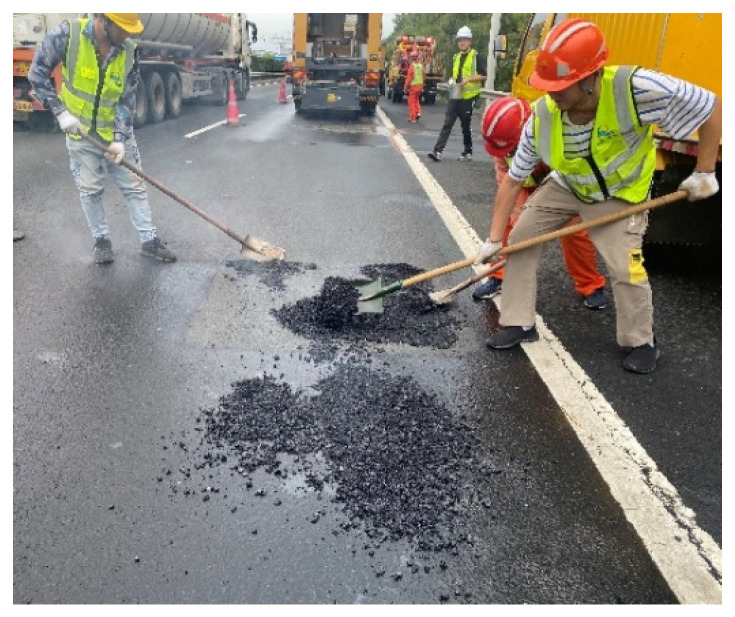
Manual milling hot pavement.

**Figure 7:**
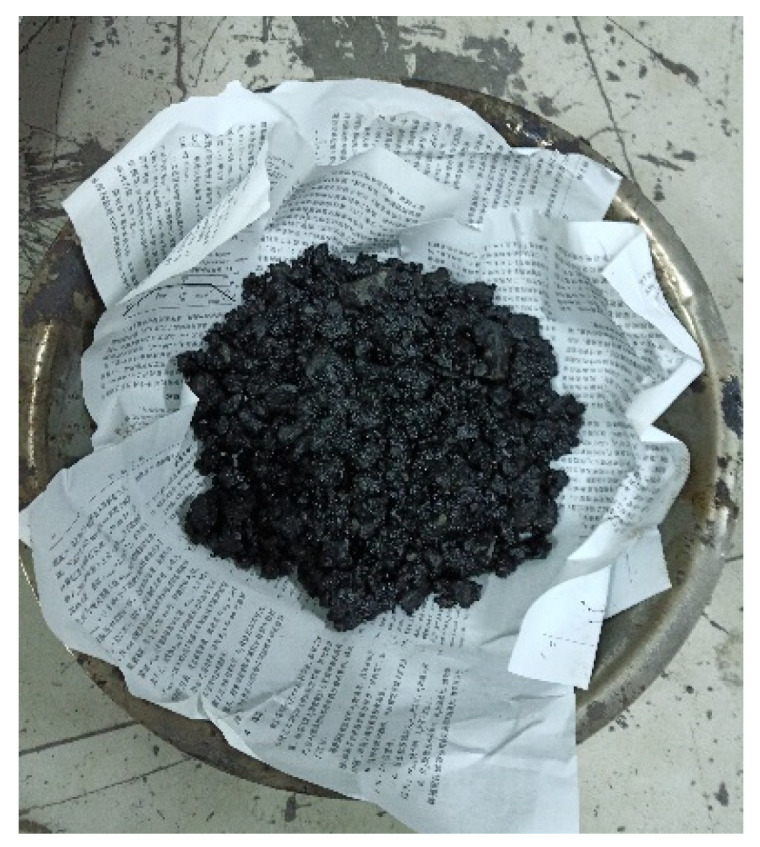
Reclaimed asphalt mixture.

The recycled asphalt pavement (RAP) used in the study was from the large maintenance engineering project of the Wuxi section in the Huning Expressway. The RAP gradation was stone matrix asphalt with a nominal aggregate size of 13.2 mm (SMA-13) located at the upper layer of the pavement structure. The original pavement was reclaimed using a hot milling technique to reduce the impact of the milling operation on the RAP gradation. Figure 5, Figure 6 and Figure 7 depict the RAP reclaiming and the on-site milling construction.

#### 2.2.2. Recycling Agent

The recycling agent of RA-102 was produced by the Subbo7t Company. The physical and mechanical properties, including the viscosity, flash point, saturated fraction content, aromatic content, viscosity ratio, and quality changes before and after the rolling thin film oven test (RTFOT), were measured with corresponding test specifications. The test results are shown in Table 1.

#### 2.2.3. Asphalt

The performance graded 76-22 SBS-modified asphalt was used in this study. The asphalt properties were tested following the Chinese specification of the “Test Procedure for Asphalt and Asphalt Mixture for Highway Engineering” (JTG E20-2011). The test results are shown in Table 2.

#### 2.2.4. Aggregate

The coarse aggregate was made of basalt with hard, clean, rough, and tough properties. The fine aggregate and mineral filler were made of limestone with properties of dry, clean, non-clumpy, and free from impurities. The technical indexes are shown in Table 3 and Table 4.

#### 2.2.5. Fiber

**Figure 8:**
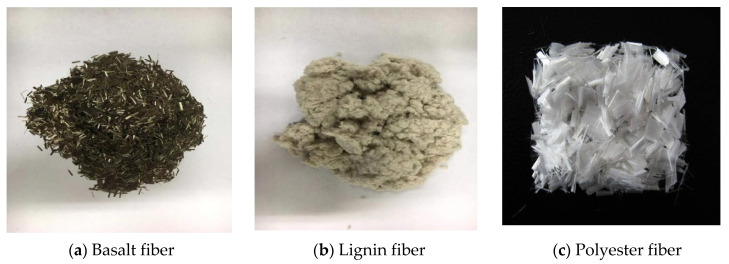
Morphology of used fibers.

Three types of fibers, including basalt fiber (BF), lignin fiber (LF), and polyester fiber (PF), were considered to create rejuvenated fiber asphalt mixtures. The basalt fiber and polyester fiber were short-cut fibers produced by Jiangsu Tianlong Basalt Continuous Fibers Co Ltd. The BF was golden brown in color, flat, and free from impurities, whereas the PF is pure white, flat, with no impurities. The lignin fiber used was the white flocculent fiber type ZZ8/1 from Riedenmei and Sons. Table 5 presents the results of the technical performance tests conducted on the three fibers, and Figure 8 displays the fiber morphology.

### 2.3. Testing of Reclaimed Asphalt Mixture

#### 2.3.1. RAP Gradation and Asphalt Content

**Figure 9:**
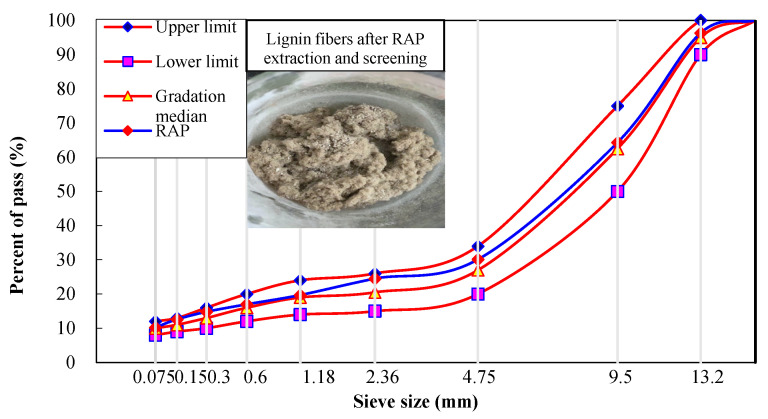
Gradation of RAP.

The old asphalt and old aggregates were recycled using the centrifugal separation process in accordance with T0722-1993 in the Chinese specification of JTG E20-2011. Trichloroethylene was used as the extraction solvent. The asphalt mixture mineral gradation test method (T0725-2000) was conducted to determine the asphalt content and aggregate gradation of the reclaimed asphalt mixture. The results show that the asphalt content in the RAP is 5.8%, and the gradation of the RAP is shown in Figure 9.

As can be seen in Figure 2, the RAP gradation is more closely aligned with the median gradation curve following the extraction and screening processes used in the layered hot milling technology. The old pavement used lignin fiber as a stabilizer, which was also extracted and shown in Figure 2. It was clear to see the quality loss and the aging phenomenon of the LF. Hence, the appropriate number of new fibers should be added to revitalize the rejuvenated SMA-13 mixture in the regeneration mix design.

#### 2.3.2. Properties of Old Asphalt

The properties of old asphalt including the penetration degree, softening point, ductility, and viscosity were tested. The test results are shown in Table 6.

It can be seen from this table that the technical indexes of the softening point and viscosity at 135 °C for the old asphalt can meet the SBS-modified asphalt specification requirements, while the values of the penetration degree and ductility are lower than the requirements. The old asphalt belongs to grade II aging, and the degree of aging is light [21].

#### 2.3.3. Dosage of Recycling Agent

Since the aged asphalt might cause negative impacts on the rejuvenated asphalt mixture, the recycling agent was thought to be able to restore part of the aged asphalt properties. The performance design method was used to determine the proper dosage of the recycling agent in the total asphalt. The dosage of the recycling agent was selected at four levels, including 4%, 6%, 8%, and 10%, respectively. General performance tests of rejuvenated asphalt were conducted. The test results are shown in Table 7.

Table 7 demonstrates that as the dosage of the recycling agent is increased, the penetration degree and ductility of rejuvenated asphalt increase, while the softening point declines. When the dosage of the recycling agent reaches 6%, the penetration degree and softening point of rejuvenated asphalt return to the performance level of the original SBS asphalt. Hence, the recycling agent dosage of 6% was chosen to formulate the hot rejuvenated asphalt mixtures.

### 2.4. Mix Proportion Design of HRAM

The HRAM was supplemented with basalt fibers (BF), lignin fibers (LF), polyester fibers (PF), and basalt–polyester compound fibers (BPCF) to evaluate the impact of various fibers on high-temperature performance and moisture susceptibility. The RAP content in the hot rejuvenated asphalt mixture for HIPR was set at 80%, while the RAP content for HMPR was set at 30%, taking into account the distinct maintenance procedures between the HIPR and the HMPR technologies.

#### Gradation Design

**Figure 10:**
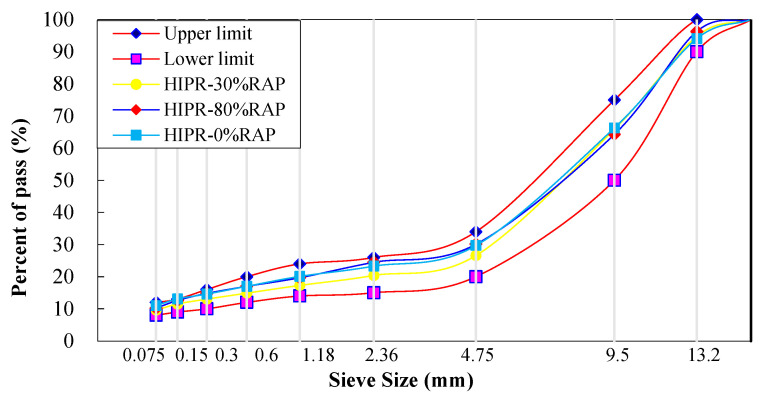
Gradation curves with three maintenance technologies.

Marshall test was carried out for new gradation design based on the requirements of SMA-13 asphalt mixture in the Chinese specification of JTG F40-2004. The designed gradation curves under the two maintenance technologies (HIPR and HMPR) were shown in Figure 10. The gradation curve of the milling resurfacing (MR) technology was also shown in this figure as a control group, and the RAP content is 0% in this group. The optimal asphalt content of original and rejuvenated fiber asphalt mixtures is shown in Table 8, and the volumetric parameters of these mixtures are listed in Table 9.

## 3. Experiments and Results

### 3.1. Hamburg Wheel Tracking Test

**Figure 11:**
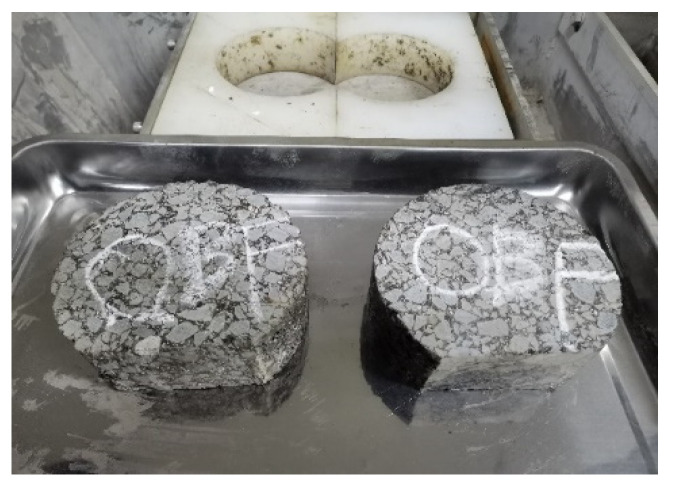
SGC specimen.

**Figure 12:**
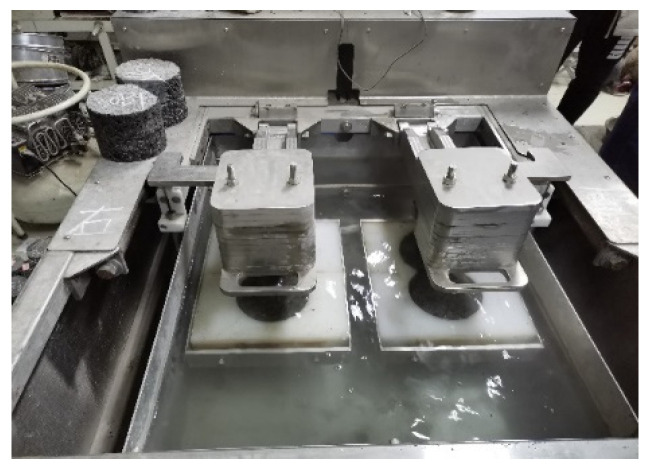
HWTT apparatus.

**Figure 13:**
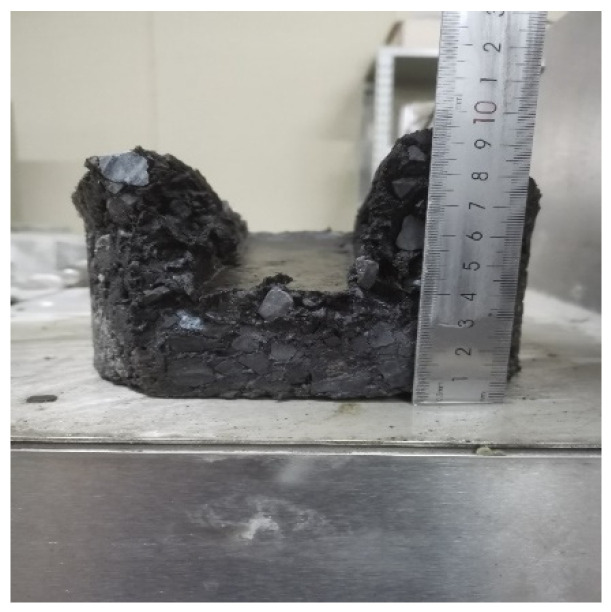
Specimen of HIPR-BF.

**Figure 14:**
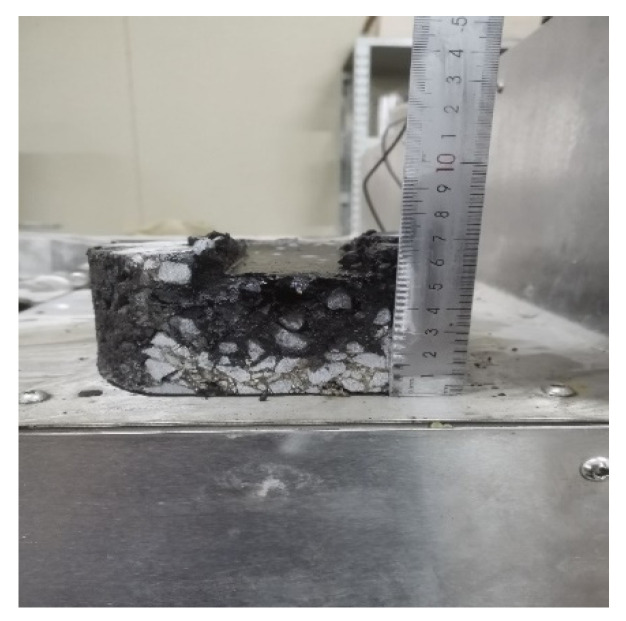
Specimen of HMPR-BF.

According to AASHTO T 324, the Superpave gyratory compactor (SGC) was selected to create cylinder specimens with a diameter of 150 mm, a thickness of 60 mm, and a target void content of 7.0 ± 0.5%. After the SGC specimen had been cooled, a wet saw was used to cut along the equidistant line so that the specimen could be embedded in the mold. The water bath temperature of the HWTT apparatus was set at 60 °C, and the specimen was submerged in water for more than 20 mm. The reciprocating motion of the vehicle load on the specimen was simulated by a steel wheel with a diameter of 203.2 ± 2.0 mm and a width of 47 ± 0.5 mm under a load of 703 ± 4.5 N. The position of the steel wheel changed sinusoidally over time, traversing the specimen 52 times per minute, and reaching a top speed of 0.305 ± 0.02 m/s at the midpoint of the specimen. The linear variable differential displacement transducer (LVDT) was used to record the rut depth at 11 points along the direction of wheel crush in real time. The HWTT apparatus and specimen are shown in Figure 11 and Figure 12. After the test was completed, the morphology of specimens such as HIPR-BF and HMPR-BF is shown in Figure 13 and Figure 14. As can be seen from these figures, there exists a big difference in the rut depths at the end of the test with distinct maintenance technologies.

### 3.2. Test Results

**Figure 15:**
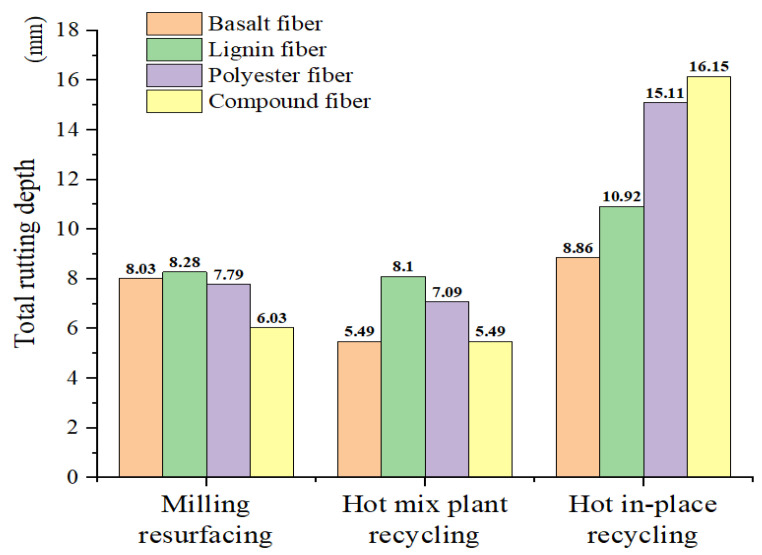
Total rutting depth at the end of HWTT.

The rut depth of eight rejuvenated asphalt mixtures and four new asphalt mixtures was recorded during the HWTT test, and the total rut depth of each mixture is shown in Figure 15. It can be seen from this figure that the HMPR asphalt mixture (i.e., 30% RAP) has the lowest rut depth when compared to the other two types of combinations, while the HIPR asphalt mixture (i.e., 80% RAP) has the maximum rut depth at the end of the test. The hydrothermal coupling performance of HWTT tends to improve and then decline with the increase in RAP materials. This is primarily caused by the modification to the aged asphalt content in the RAP materials, which increases the early-stage rutting resistance of rejuvenated asphalt mixtures. However, the ability of rejuvenated asphalt mixtures to resist moisture damage is insufficient to support cyclic heavy loads when the RAP content goes above a certain point. Based on three maintenance techniques, the BF combinations show better rutting resistance than the other three types of rejuvenated fiber asphalt mixtures.

Since the HWTT can simultaneously characterize the rutting resistance and water stability of the asphalt mixture, many researchers have tried to find the feature points of the HWTT curve to study its performance at different periods. There are three main stages for the specimen under wheel load, including the post-compaction stage (PCS), the creep stage (CS), and the stripping stage (SS) [22], as shown in Figure 15. The specimen in the PCS is subjected to a high compression force given by the HWTT wheels, and the air voids are post-compacted. Hence, the rut depth in the PCS changes at a faster rate and for a shorter period as the number of wheels increases. At the CS, the rut depth increases steadily, and materials in the specimen tend to have shear flow and continue to produce rutting damage under the wheel load. At the SS, the asphalt mixture is subjected to the double effects of wheel load as well as high-temperature moisture damage; part of the asphalt loses its adhesive bonds, the asphalt mixture begins to peel off, and the rutting and stripping of the specimen are accelerated.

**Figure 16:**
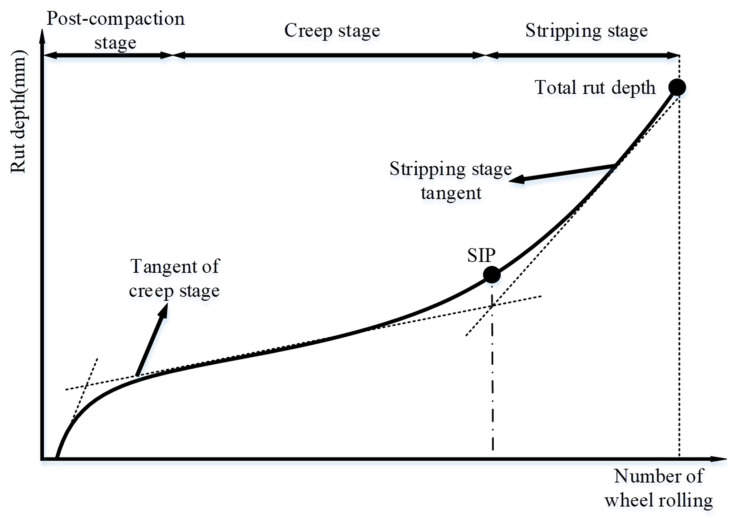
Schematic diagram of a conventional HWTT curve.

Currently, the total rut depth (TRD) and stripping inflection point (SIP) are widely recognized as HWTT performance indicators. The SIP is the inflection point from CS to SS, which is commonly recognized as the intersection of the two straight lines fitted by CS points and SS points, as seen in Figure 16. In a sense, the value of SIP is greatly affected by the slope of two fitting lines. However, the slope of two fitting lines in the CS and SS seems not to be unified. Hence, a new methodology or mathematical model is needed to be developed to give a higher accuracy of SIP determination.

The HWTT curve is made up of two parts, the first of which has a negative curvature and is followed by the other, which has a positive curvature, according to the findings of earlier studies on rutting prediction models conducted by our research group [14] and Fan Y et al. [22]. This curve can be completely represented by Equation (1).

(1) $$RD=\rho *{\left[ln\left(\frac{N_{ult}}{N-N_{0}}\right)\right]}^{-\frac{1}{\beta }}$$

where N = the number of load cycles; RD = the rut depth at a certain number of load cycles (mm); $N_{0}$ = the number of load cycles where rut depth occurs; ρ, $N_{ult}$, and β = model coefficients.

**Figure 17:**
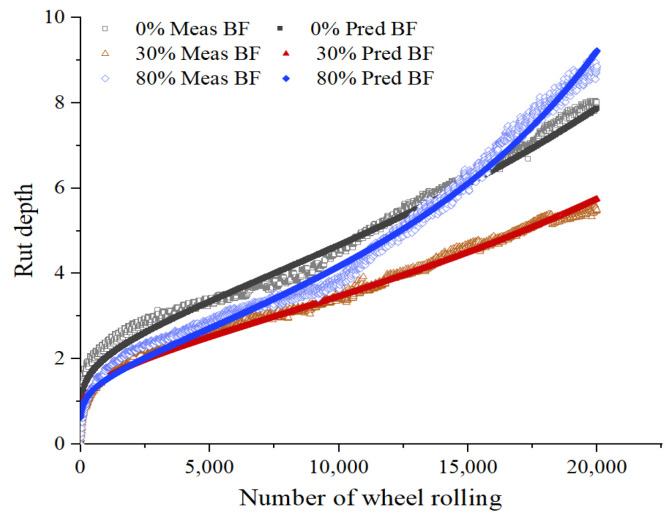
HWTT curve of BF asphalt mixture.

**Figure 18:**
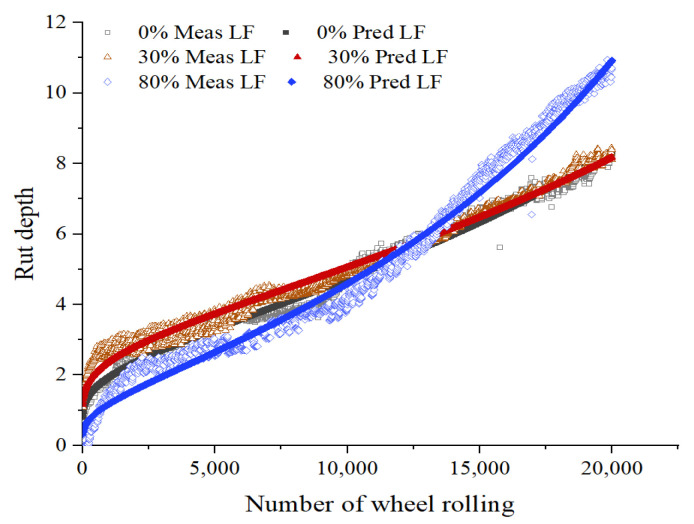
HWTT curve of LF asphalt mixture.

**Figure 19:**
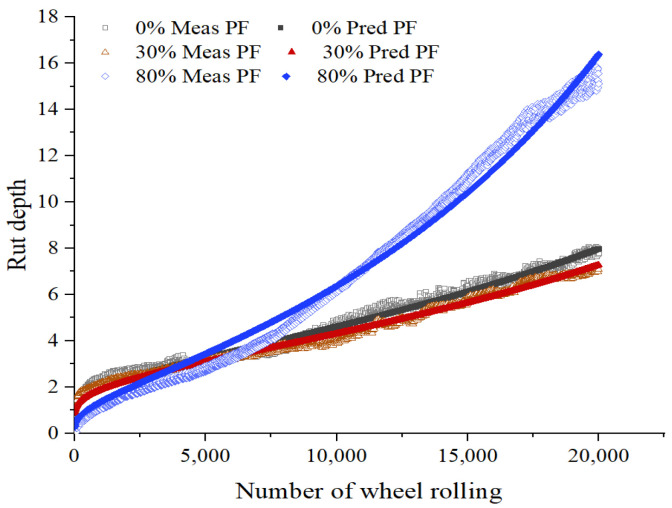
HWTT Curve of PF Asphalt Mixture.

**Figure 20:**
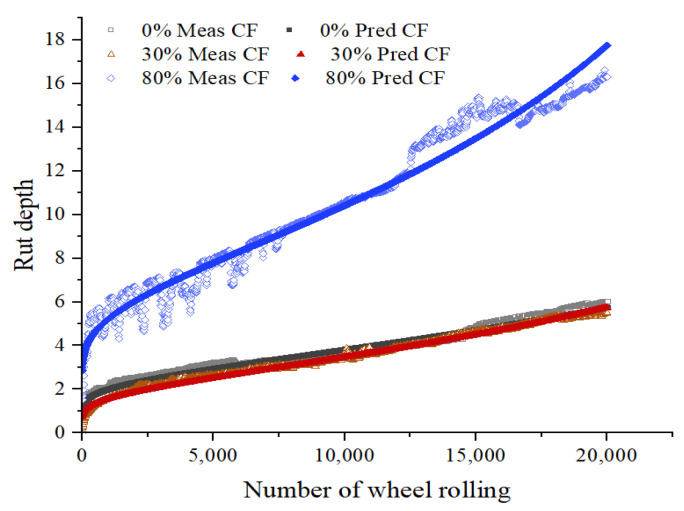
HWTT curve of BPCF asphalt mixture.

Using the above formula to fit the HWTT data under the three maintenance technologies, the fitting curves are shown in Figure 17, Figure 18, Figure 19 and Figure 20, and the relevant fitting parameters are shown in Table 10. From this figure, it can be seen that all four fiber asphalt mixtures have been wheeled 20,000 times. The HIPR asphalt mixtures show good resistance to high-temperature rutting in the post-compaction and creep stages, but they present poor water stability in the stripping stage.

There are three feature points in the HWTT rutting curve, which can be used to separate the post-compaction stage, the creep stage, and the stripping stage. Finding the feature points in the curve fitting model can better characterize the HWTT results. The first derivative of Equation (1) is taken to obtain the slope of the HWTT curve, as given in Equation (2).

(2) $$RD^{\prime }=\frac{\rho }{N*\beta }{\left(ln\frac{N_{ult}}{N}\right)}^{-\frac{1}{\beta }-1}$$

When the curvature of the HWTT curve turns from negative to positive, the second inflection point can be determined. The second derivative of Equation (1) is given as,

(3) $$RD^{\prime \prime }=-\frac{\rho }{\beta *N^{2}}\left[{\left(ln\frac{N_{ult}}{N}\right)}^{-\frac{1}{\beta }-1}+\left(-\frac{1}{\beta }-1\right){\left(ln\frac{N_{ult}}{N}\right)}^{-\frac{1}{\beta }-2}\right]$$

When the second derivative is set to zero, two solutions exist in Equation (3).

$$x_{1}=N_{ult},\;\;x_{2}=\frac{N_{ult}}{e^{1+\frac{1}{\beta }}}$$

**Figure 21:**
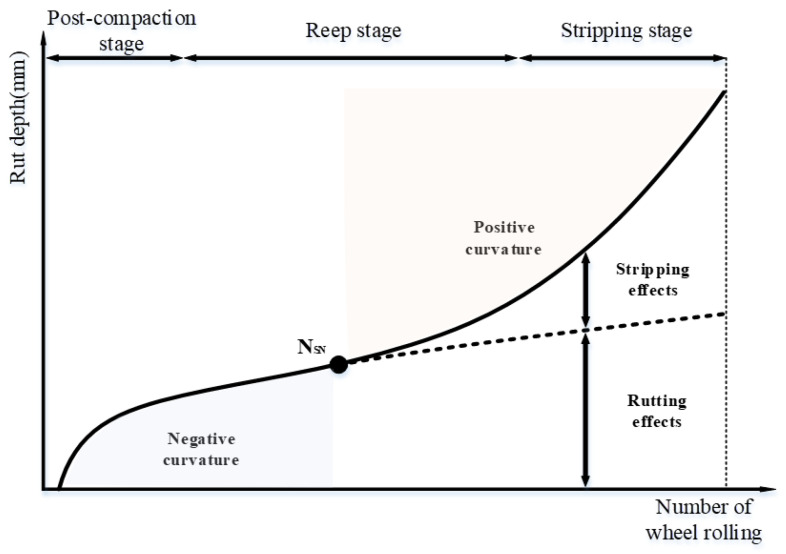
Intersection from positive curvature to negative curvature.

**Figure 22:**
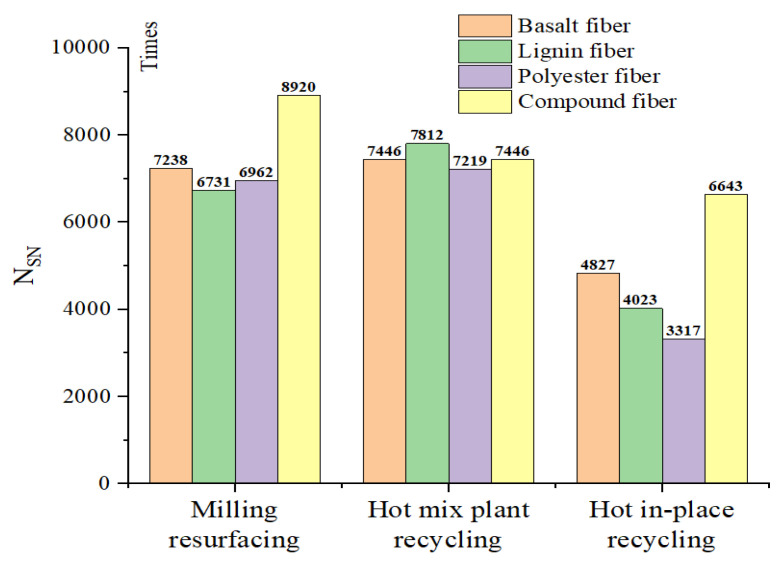
$N_{SN}$ of each fiber asphalt mixture.

Since $N_{ult}$ is much larger than 20,000 loads, the unique solution of $\;x_{2}$ is taken. This solution is the intersection (i.e., $N_{SN}$) from the positive curvature to the negative curvature, as shown in Figure 21. The value of $N_{SN}$ for each asphalt mixture is shown in Figure 22. In front of the point of $N_{SN}$, the adhesion between the aggregate and asphalt in the specimen is good, and the specimen is in the post-compaction stage with a positive curvature. After the point of $N_{SN}$, the rutting curve enters the negative curvature phase. The asphalt adhesion performance decreases when the asphalt mixture starts to produce rapid damage, and the rut depth of the specimen is accelerated by the moisture effect.

The intersection point (i.e., $N_{SN}$) manifests itself earlier as the RAP content increases, indicating that the RAP is more moisture-sensitive than new asphalt mixtures. The RAP in the mixture has the potential to accelerate moisture damage, causing the mixture to reach the stripping stage earlier.

To distinguish the damage to the asphalt mixture under the effects of high temperature and water immersion, the HWTT curve before the point of $N_{SN}$ is fitted by Equation (4), and the schematic diagram is shown in Figure 18. The curve fitting parameters were obtained, as shown in Table 11.

(4) $$RD=A\times NP^{B}$$

**Figure 23:**
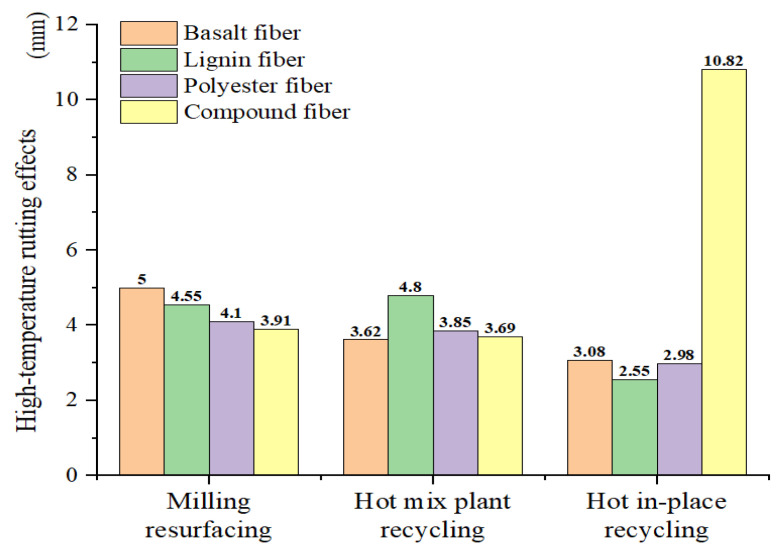
High-temperature rutting effects of fiber asphalt mixtures.

**Figure 24:**
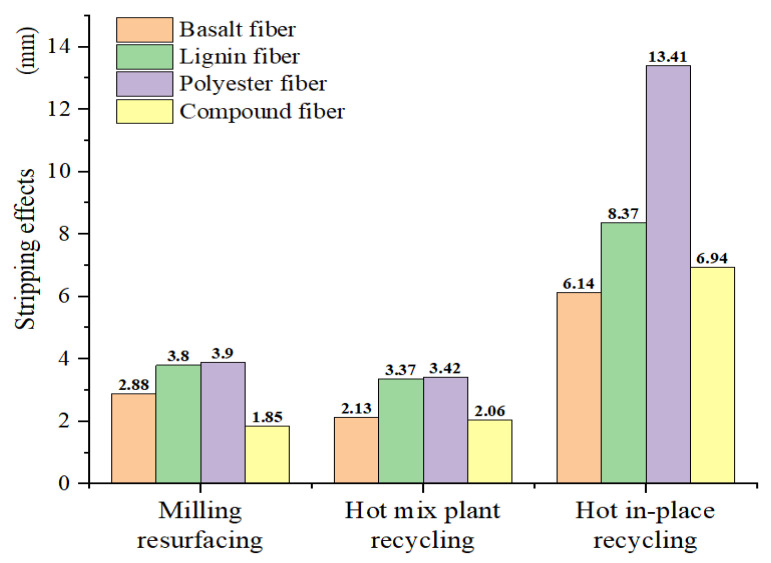
Stripping effects of fiber asphalt mixtures.

The fitting curves can be extended to the end of the test to obtain the high-temperature rutting effects in the HWTT. The rut depth at 20,000 loads calculated by Equation (4) was obtained, as shown in Figure 23. The stripping effects can be obtained by using the total rut depth minus the high-temperature rutting effects, which are expressed in Figure 18. The results of the rut depth related to the stripping effects at 20,000 loads are shown in Figure 24. Figure 23 shows that the high-temperature rutting performance decreases with the increase in the RAP content in terms of single-doped fiber asphalt mixtures, and the LF asphalt mixture in the HIPR process performed the best. Figure 24 shows that the stripping resistance displays a contrary tendency compared with high-temperature rutting resistance. The asphalt mixture with basalt-polyester compound fibers in the MR process has the best performance.

**Figure 25:**
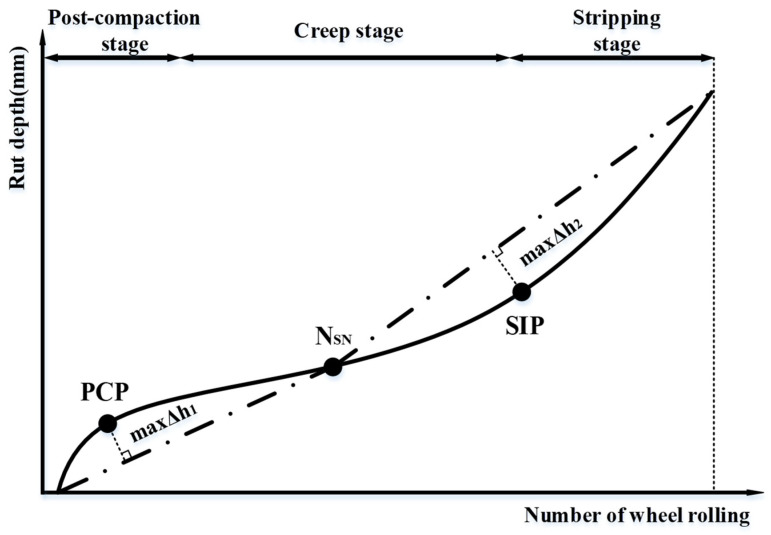
Schematic diagram of PCP and SIP redefinition.

From the above study, it is found that the inflection point of $N_{SN}$ can be obtained by setting the second derivative to zero in the three-stage curve fitting model. However, the other two critical points separate the three stages of the development of the HWTT curve, which cannot be determined directly from this model. Hence, the concept of a stationary point in the HWTT curve is introduced to mathematically redefine the post-compaction critical point and the stripping critical point. Take the BF asphalt mixture in the MR process as an example, connect the $N_{SN}$ with the curve starting point to find the first line segment: $y_{1}={5.44\;\times \;10}^{-4}x,\;\left(0\;\leq \;x\;\leq \;7238.3\right)$. Then, connect the $N_{SN}$ with the point at end of the test to obtain the second line segment: $y_{2}={3.09\;\times \;10}^{-4}x+1.71,\;(7238.3\;\leq \;x\;\leq \;20000)$. Form the vertical line between the HWTT curve and the two line segments to obtain the maximum value of $\Delta h_{1}$ and $\Delta h_{2}$. The loading cycle at the maximum value of $\Delta h_{1}$ is defined as the first stationary point (i.e., PCP) between the post-compaction stage and the creep stage. Likewise, the loading cycle at the maximum value of $\Delta h_{2}$ is defined as the second stationary point (i.e., SIP) between the creep stage and the stripping stage. A schematic diagram of the stationary point is shown in Figure 25. The corresponding line segments and three feature points (i.e., first stationary point, inflection point, and second stationary point) are obtained in this method for all HWTT curves of asphalt mixtures, as shown in Table 12.

As can be seen from Table 12, the HIPR asphalt mixtures pass in the creep stage at around 500 to 600 cycles of the wheel rolling with a low value of rut depth. The MR asphalt mixtures and HMPR asphalt mixtures entered the creep stage at around 800 to 950 cycles of wheel millings. The results show that the PCP of the HIPR asphalt mixture is earlier than that of the MR asphalt mixture and the HMPR asphalt mixture. However, the SIP seems to have a reverse trend in these mixtures. Compared with the new definition of SIP and the conventional calculation of SIP, it is found that this new method can provide more scientific theoretical support for the HWTT results for different asphalt mixtures. The PCP and SIP calculation methods can provide a theoretical basis for decision-making in maintenance projects.

## 4. Comprehensive Analysis of Cost and Performance

### 4.1. Economic Cost Analysis

The economic benefit is one of the key factors which should be also considered. The costs incurred under different maintenance methods are different, and the cost is an unavoidable problem in the practical application of the project. By constructing an economic model to analyze the costs under the three maintenance methods, it is possible to guide the concrete implementation of maintenance works.

The cost of the asphalt pavement maintenance process can be divided into four parts, namely, the milling cost, mixing cost, paving and rolling cost, and transportation cost [23]. To intuitively reflect the economic effects, the unit of price is standardized in this paper as Yuan (CNY) per ton and the expression of economic cost is shown in Equation (5).

(5) $$O=\overset{n}{\underset{i=1}{\sum }}M_{i}+\overset{m}{\underset{i=1}{\sum }}C_{i}+\overset{l}{\underset{i=1}{\sum }}P_{i}+\overset{g}{\underset{i=1}{\sum }}T_{i}$$

where M is the milling cost, C is the asphalt mixture and mixing cost, P is the paving and rolling cost, T is the transportation cost.

The asphalt mix and mixing costs are modeled as Equation (6).

(6) $$C=\left(C_{as}P_{as}+C_{ag}P_{ag}+C_{fi}P_{fi}\right)\times \left(1-R_{p}\right)+R_{p}\times C_{ra}+C_{mi}$$

where $C_{as}$ is the cost per ton of asphalt, $C_{ag}$ is the cost per ton of aggregates, $C_{fi}$ is the cost per ton of fiber, $C_{ra}$ is the cost per ton of regeneration agent, $C_{mi}$ is the mixing cost per ton if asphalt mixture; $P_{as}$ is the asphalt ratio, $P_{ag}$ is the aggregate content, $P_{fi}$ is the fiber dosage, and $R_{p}$ is the RAP content. The calculation models for milling, paving and rolling, and transportation are simply cumulative and will not be repeated here.

In the MR maintenance process, the RAP content is zero percent, and there is a regeneration agent cost. The transportation cost only includes one-way transportation of the new asphalt mixture, so the cost function is given in Equations (7) and (8).

(7) $$O_{re}=M+\overset{3}{\underset{i=1}{\sum }}C_{i}+P+T$$

(8) $$C_{re}=C_{as}P_{as}+C_{ag}P_{ag}+C_{fi}P_{fi}+C_{mi}$$

In the HMPR maintenance process, the RAP content is 30%. The transportation cost includes the round-trip cost of transporting the old asphalt mixture to the mixing plant and the recycled asphalt mixture back to the construction site. The cost calculation model is Equations (9) and (10).

(9) $$O_{pl}=M+\overset{4}{\underset{i=1}{\sum }}C_{i}+P+\overset{2}{\underset{i=1}{\sum }}T_{i}$$

(10) $$C_{pl}=\left(C_{as}P_{as}+C_{ag}P_{ag}+C_{fi}P_{fi}\right)\times 70\%+30\%\times C_{ra}+C_{mi}$$

In the HIPR maintenance process, the RAP content is 80%. Since the HIPR equipment is used, the fuel consumption and vehicle rental cost of the HIPR equipment are used to replace the separate costs of milling and paving, and the model is shown in Equations (11) and (12).

(11) $$O_{ip}=\overset{4}{\underset{i=1}{\sum }}C_{i}+\overset{3}{\underset{i=1}{\sum }}T_{i}$$

(12) $$C_{ip}=\left(C_{as}P_{as}+C_{ag}P_{ag}+C_{fi}P_{fi}\right)\times 20\%+80\%\times C_{ra}+C_{mi}$$

**Figure 26:**
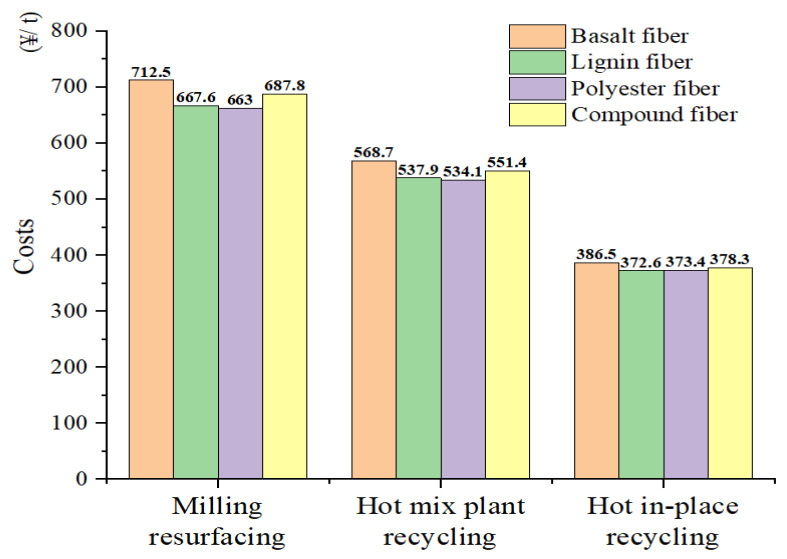
Maintenance cost of each maintenance process.

Through the on-site investigation of the actual project, it is assumed that the asphalt mixing plant is 30 km away from the construction site. The cost list under the three maintenance processes and the cost details of each maintenance stage are obtained, as shown in Table 13 and Table 14. The final costs for the three maintenance processes are shown in Figure 26.

As can be seen from Figure 26, with the incorporation of RAP, the costs of the three maintenance processes are listed in descending order: MR > HMPR > HIPR. The cost of a new asphalt mixture in the HIPR process is only 29% of that in the MR process. The HIPR process is a little expensive in terms of equipment leasing and fuel consumption compared with the MR process. In general, the HIPR process cost only accounts for 55% of the MR process cost. The HMPR process is similar to the MR process, but due to the saving materials in the new asphalt mixture, the cost of the HMPR process is 80% of the MR process. As for fiber types, the BF is more expensive than the LF and PF when making fiber asphalt mixtures. However, the asphalt absorption for the LF is much larger than that of the BF and PF, hence the asphalt content for the LF asphalt mixture is relatively high. The cost of asphalt is greater than the cost of aggregate, making the cost of the LF asphalt mixture slightly higher than that of the PF asphalt mixture.

### 4.2. Comprehensive Benefit Analysis Based on the Grey Relational Method

The above study investigated the resistance to HWTT test performance and the respective economic benefit of asphalt mixture with different types of fibers incorporated in asphalt pavements under three maintenance processes. The MR process is found to be more resistant to HWTT rutting with a higher cost, and the HIPR process is found to be less resistant to moisture damage in the stripping stage but at a significant price advantage. Considering the need for test performance and economic cost in practical engineering, this study extracts the main factors affecting performance and cost through grey correlation analysis. Then, the optimal maintenance process and fiber type are selected with the highest comprehensive benefit.

The grey correlation analysis is considered as a systematic approach to analyzing finite and irregular data. It can create a grey series that gives a holistic view and comparison to determine the optimal solution. In this study, 12 kinds of asphalt mixtures with different maintenance processes and fiber types are selected as the scheme. The feature points in the HWTT curve and the cost for the corresponding maintenance process are used as the impact factors, and the optimal scheme is obtained by analyzing the influence of each factor on the comprehensive benefit. Asphalt mixtures of different types can be used to construct scheme A, where $A=\left\{A_{1},A_{1},\;\ldots ,\;A_{m}\right\}$, m=12. For each type of asphalt mixture, the total rut depth (TRD), $N_{SN}$, rut depth for high-temperature rutting effect, rut depth for stripping effect, rut depth and loading cycle at PCP, rut depth and loading cycle at SIP, and the cost of maintenance process are taken as scheme B, where $B=\left\{B_{1},B_{1},\;\ldots ,\;B_{n}\right\}$, n = 9. Constructing matrix X from A and B, in which $X={\left(x_{ij}\right)}_{m\times n}$, and $x_{ij}$ is the jth test indicator for the ith scheme. The column preference matrix X is given as,

$$X=\left(\begin{array}{l} 8.033\;7238.3\;5.001\;2.884\;810\;1.95\;14500\;5.96\;712.5\\ 8.284\;6730.8\;4.546\;3.801\;780\;1.40\;14325\;6.11\;667.6\\ 7.785\;6961.8\;4.101\;3.899\;790\;1.43\;14410\;5.95\;663.0\\ 6.034\;8919.9\;3.909\;1.851\;950\;1.63\;18120\;5.35\;687.8\\ 5.490\;7446.2\;3.623\;2.125\;810\;1.16\;14615\;4.41\;568.7\\ 8.104\;7811.9\;4.804\;3.370\;820\;1.81\;14780\;6.41\;537.9\\ 7.093\;7219.1\;3.852\;3.424\;800\;1.39\;14515\;5.50\;534.1\\ 5.490\;7446.3\;3.688\;2.062\;810\;1.16\;14610\;4.41\;551.4\\ 8.861\;4827.4\;3.084\;6.140\;550\;1.00\;13960\;5.64\;386.5\\ 10.92\;4022.6\;2.553\;8.372\;570\;0.63\;13260\;6.17\;372.6\\ 15.11\;3316.7\;3.981\;13.41\;505\;0.63\;12930\;8.56\;373.4\\ 16.15\;6643.3\;10.82\;6.936\;600\;4.34\;14530\;13.4\;378.3 \end{array}\right)$$

In order to facilitate calculation and comparison, the indicators need to be dimensionless. For the larger and better indicators, the dimensionless formula is: $y_{ij}=\frac{max\left(x_{j}\right)-x_{ij}}{max\left(x_{j}\right)-min\left(x_{j}\right)}$. For smaller and better indicators, the dimensionless formula is: $y_{ij}=\frac{x_{ij}-min\left(x_{j}\right)}{max\left(x_{j}\right)-min\left(x_{j}\right)}$, of which, j=1,2, …, n. Where $y_{ij}$ is the value of the jth index of the ith scheme, $max\left(x_{j}\right)\;$ and $min\left(x_{j}\right)$ represent the maximum and minimum values in indicator j, respectively. After dimensionless processing, the matrix Y can be obtained.

$$Y=\left(\begin{array}{l} 0.76\;0.70\;0.70\;0.91\;0.69\;0.64\;0.30\;0.83\;0.00\\ 0.74\;0.61\;0.76\;0.82\;0.62\;0.79\;0.27\;0.81\;0.13\\ 0.78\;0.65\;0.81\;0.82\;0.64\;0.78\;0.29\;0.83\;0.15\\ 0.95\;1.00\;0.84\;1.00\;1.00\;0.73\;1.00\;0.90\;0.07\\ 1.00\;0.74\;0.87\;0.98\;0.69\;0.86\;0.32\;1.00\;0.42\\ 0.75\;0.80\;0.73\;0.87\;0.71\;0.68\;0.36\;0.78\;0.51\\ 0.85\;0.70\;0.84\;0.86\;0.66\;0.80\;0.31\;0.88\;0.52\\ 1.00\;0.74\;0.86\;0.98\;0.69\;0.86\;0.32\;1.00\;0.47\\ 0.68\;0.27\;0.94\;0.63\;0.10\;0.90\;0.20\;0.86\;0.96\\ 0.49\;0.13\;1.00\;0.44\;0.15\;1.00\;0.06\;0.80\;1.00\\ 0.10\;0.00\;0.95\;0.00\;0.00\;1.00\;0.00\;0.54\;1.00\\ 0.00\;0.59\;0.00\;0.56\;0.21\;0.00\;0.31\;0.00\;0.98 \end{array}\right)$$

The $H_{j}$ is defined as the entropy of the *j*th indicator, which can be expressed as $H_{j}=-\frac{\sum_{i=1}^{m}f_{ij}ln\left(f_{ij}\right)}{lnm}$, j=1,2, …, n, in which, $f_{ij}=\frac{y_{ij}}{\sum_{i=1}^{m}y_{ij}}$. In order to make sense of $ln\left(f_{ij}\right)$ in the entropy formula, it is assumed that $f_{ij}=0$, $ln\left(f_{ij}\right)=0$. Thus, the $H_{j}$ can be obtained. $H_{j}=\left[0.93\;0.93\;0.96\;0.95\;0.91\;0.96\;0.90\;0.96\;0.88\right]$.

The entropy weight $W_{j}$ can be expressed as $W_{j}=\frac{1-H_{j}}{\sum_{j=1}^{n}\left(1-H_{j}\right)}$, and the entropy weight matrix $W_{j}$ is given as,

$$W_{j}=diag\left\{0.11,\;0.11,\;0.06,\;0.07,\;0.15,\;0.06,\;0.17,\;0.06,\;0.20\right\}$$

Transforming matrix Y to attribute matrix R, which can be expressed as $R=Y\times W_{j}$.

$$R=\left(\begin{array}{l} 0.081\;0.078\;0.043\;0.068\;0.102\;0.041\;0.052\;0.053\;0.000\\ 0.079\;0.068\;0.046\;0.062\;0.092\;0.050\;0.046\;0.052\;0.026\\ 0.084\;0.073\;0.049\;0.061\;0.095\;0.050\;0.049\;0.053\;0.029\\ 0.101\;0.112\;0.051\;0.074\;0.149\;0.046\;0.170\;0.057\;0.015\\ 0.107\;0.082\;0.053\;0.073\;0.102\;0.054\;0.055\;0.064\;0.084\\ 0.081\;0.090\;0.044\;0.065\;0.106\;0.043\;0.061\;0.050\;0.103\\ 0.091\;0.078\;0.051\;0.064\;0.099\;0.050\;0.052\;0.056\;0.105\\ 0.107\;0.082\;0.052\;0.072\;0.102\;0.054\;0.055\;0.064\;0.095\\ 0.073\;0.030\;0.057\;0.047\;0.015\;0.057\;0.034\;0.055\;0.191\\ 0.052\;0.014\;0.061\;0.032\;0.022\;0.063\;0.011\;0.052\;0.200\\ 0.010\;0.000\;0.058\;0.000\;0.000\;0.063\;0.000\;0.035\;0.199\\ 0.000\;0.066\;0.000\;0.042\;0.032\;0.000\;0.053\;0.000\;0.196 \end{array}\right)$$

Then, the ideal point of the matrix (P) can be selected and expressed as $P=\left[p_{1},p_{2},\;\ldots ,\;p_{n}\right]$. In which, $p_{j}=max\left\{r_{ij}|i=1,\;2,\;\ldots ,\;m;j=1,\;2,\;\ldots ,\;n\right\}$.

$$P=\left[0.107\;0.112\;0.061\;0.074\;0.149\;0.063\;0.170\;0.064\;0.200\right]$$

The distance to the ideal point for each scenario (L) can be calculated, where $L=\left[l_{1},l_{2},\;\ldots ,\;l_{m}\right]$, of which, $l_{i}=\sqrt{\overset{n}{\underset{j=1}{\sum }}{\left(r_{ij}-p_{j}\right)}^{2}}$, i=1, 2,…,m.

$$L=\left[\begin{array}{c} 0.059\;0.052\;0.049\;0.035\\ 0.030\;0.025\;0.027\;0.027\\ 0.045\;0.056\;0.079\;0.054 \end{array}\right]$$

Therefore, the ordering of the 12 scenarios is: $L_{6}<L_{7}=L_{8}<L_{5}<L_{4}<L_{9}<L_{3}<L_{2}<L_{12}<L_{10}<L_{1}<L_{11}$. According to the grey relational analysis data, it can be seen that the HMPR process has the highest comprehensive benefit among the three maintenance processes. The HMPR asphalt mixture with LF has the best performance, followed by that of the PF and BPCF. The MR fiber asphalt mixture has a similar trend. In the HIPR maintenance process, BF is the first choice. Combined with the HWTT test data, it can be seen that the HIPR maintenance process has poor stripping resistance, while the performance can be significantly improved with the addition of BF in the HIPR mixture. Therefore, the BF can be used as the preferred reinforcement and toughening material for asphalt mixtures with a high RAP dosage.

## 5. Conclusions

This study proposes an innovative method to analyze the performance and economic aspects of rejuvenating fiber asphalt mixtures in high-temperature moisture susceptibility. Under different maintenance processes, Hamburg wheel tracking tests are conducted to investigate the rutting and stripping effects of rejuvenated fiber asphalt mixtures. Major conclusions can be drawn as follows:

(1) The best performance in terms of resistance to hydrothermal coupling effects is achieved at the HMPR maintenance process, with the asphalt mixtures incorporating the BF and BPCF. Both of these two mixtures have a TRD of about 5.49 mm. The high-temperature rutting performance of asphalt mixtures improves with the increase in RAP content. The HIPR asphalt mixture with the LF has the best high-temperature rutting performance, with a rutting depth of 2.55 mm at a 20,000 cycle. The moisture damage resistance is negatively correlated with the high-temperature rutting performance. The asphalt mixture with the MR process is the best, and the rutting depth under the stripping effect is 1.85 mm.

(2) The HWTT curve fitting model considers the positive and negative curvature of the curve in two separate phases. The starting and ending points of the HWTT curves are connected with the intersection point to obtain two line segments. The unique solution of the PCP and SIP points is obtained by finding the maximum vertical distances between the HWTT curve and two line segments. This method can give a generalized approach to determining the value of stripping inflection points mathematically. The results show that the three feature points of the PCP, N_SN, and SIP are the earliest for the HIPR maintenance process, while these points for asphalt mixtures with the BF and BPCF under the MR maintenance process are the latest, at 950,892,018,120 loading cycles, respectively.

(3) The economic model of three maintenance processes was established. The results found that the cost of the MR maintenance process is the most expensive, while the asphalt mixtures incorporated with the BF increase the cost of maintenance to 712.5 ¥/t. The HIPR maintenance process is more expensive in terms of fuel consumption and equipment leasing, but the cost of raw materials such as aggregates and asphalt is significantly lower. The cost of the HIPR maintenance process is only 55% of the cost of the MR maintenance process.

(4) The performance and economic benefits of 12 rejuvenated asphalt mixtures were investigated by grey correlation analysis. Nine indicators including TRD, N_SN, the RD of high-temperature rutting effect, the RD of stripping effects, RD and N at PCP, RD and N at SIP, and maintenance cost are used as the impact indicators. The results indicate that the HMPR maintenance process is the most efficient within the three maintenance processes, and the HMPR maintenance process with LF is the best choice. In the HIPR maintenance process, the incorporation of BF improves the HWTT performance of the asphalt mixture.

Recycling and recovering are the main trends in the future development of the pavement maintenance industry. In this study, the HWTT curve analysis method was improved on the background of three maintenance technologies. The proposed approach can be used to identify three feature points to better control rutting resistance and moisture susceptibility. A comprehensive benefits analysis combining performance and economic benefits can be investigated. However, a full life cycle analysis is still lacking and should be focused on in future research.

## Figures and Tables

**Table 1:** Technical index of Subtherm RA-102 Recycling agent.

Technical Index	RA-102	National Normative Criterion	Test Method
90 °C Viscosity, cP	4000	-	T0619
Flash point/°C	248	≥220	T0633
Saturated fraction content/%	25.6	≤30	T0618
Aromatic content/%	53	≥30	T0618
Viscosity ratio before and after RTFOT	1.34	≤3	T0610
Quality changes before and after RTFOT/%	1.02	≤4%	T0603

**Table 2:** SBS-modified asphalt (PG76-22) technical index.

Test Items		Specification Requirement	Test Result	Test Method
Penetration degree (25 °C)/0.1 mm		60~80	71	T0604
Softening point/°C		≥55	64	T0606
Ductility (5 cm/min, 5 °C)/cm		≥30	48	T0605
Penetration index (PI)		−0.4~1.0	0.5	T0604
Segregation (softening point difference)/°C		≤2.5	1.4	T0661
Elasticity recovery (25 °C)/%		≥65	76	T0662
Residue after RTFOT	Quality change/%	±1.0	−0.08	T0610
	Penetration ratio/%	≥60	86	T0604
	15 °C Residual ductility/cm	≥20	37	T0605

**Table 3:** Technical indexes of coarse and fine aggregates.

Aggregate Type		Apparent Specific Gravity	Gross Volume Relative Density
Basalt aggregate	1#	2.953	2.908
	2#	2.962	2.902
	3#	2.902	2.853
Limestone aggregate	4#	2.704	/

**Table 4:** Technical indexes of mineral fillers.

Targets of Test		Test Result	Specification Requirement	Test Method
Water content/%		0.3	≤1	Drying method
Relative density		2.654	≥2.5	T0352
Appearance		no agglomerates	no agglomerates	/
Hydrophilic coefficient		0.63	<1	T0353
Particle size range	<0.6 mm	100	100	T0351
	<0.15 mm	92.6	90~100	
	<0.075 mm	92.2	75~100	

**Table 5:** Technical performances of fibers.

Technical Performances	Basalt Fiber	Lignin Fiber	Polyester Fiber
Diameter (μm)	16.23	0.045	16.58
Density (g/cm^2^)	2.71	0.82	0.95
Tensile strength (MPa)	2785	265	1010
Breaking elongation (%)	5.72	17.26	34.72

**Table 6:** Old asphalt properties.

Technical Index	Old Asphalt	SBS-Modified Asphalt Requirements	Test Method
Penetration degree (25 °C)/0.1 mm	39	50–80	T0604
Softening point/°C	69	>60	T0606
Ductility/cm	7.8	>30	T0605
Viscosity (135 °C)/Pa·s	2.33	≤3	T0613

**Table 7:** Test results of rejuvenated asphalt.

Technical Index	Recycling Agent Dosage (%)					New SBS-Modified Asphalt	Test Method
	0	4	6	8	10		
Penetration degree (25 °C)/0.1 mm	39	60	68	74	78	71	T0604
Softening point/°C	69	65	63	61	56	64	T0606
Ductility/cm	7.8	22.4	28.6	31.4	34.6	48	T0605

**Table 8:** Fiber dosage and asphalt content under three maintenance processes.

Number	Grading Type	Fiber Types	Fiber Dosage/‰	Optimal Asphalt Content/%
1	MR SMA-13	LF	3	6.0
2	MR SMA-13	BF	3	5.8
3	MR SMA-13	PF	3	5.8
4	MR SMA-13	BPCF	1.5/1.5	5.8
5	HMPR SMA-13	LF	3	6.1
6	HMPR SMA-13	BF	3	5.9
7	HMPR SMA-13	PF	3	5.9
8	HMPR SMA-13	BPCF	1.5/1.5	5.9
9	HIPR SMA-13	LF	1	6.0
10	HIPR SMA-13	BF	3	6.0
11	HIPR SMA-13	PF	3	6.0
12	HIPR SMA-13	BPCF	1.5/1.5	6.0

**Table 9:** Volumetric parameters of aggregate gradations.

Volume Parameters	MR-BF	MR-LF	HMPR-BF	HMPR-LF	HIPR-BF	HIPR-LF	Specification Requirements
VV/%	4.3	3.8	4.1	4.0	4.1	4.0	3~4.5
VMA/%	16.8	17.2	17.1	17.3	17.5	17.5	≥16.5
VFA/%	74.4	78.0	76.0.	76.9	76.9	78.3	70~85
Stability/kN	11.50	11.93	12.05	11.5	12.45	11.28	≥6
Gross volume relative density	2.514	2.507	2.520	2.507	2.518	2.521	-
Maximum theoretical relative density	2.627	2.605	2.628	2.611	2.623	2.621	-

**Table 10:** HWTT fitting parameters of each asphalt mixture.

Mixture Type	HWTT in Wet Condition			R-Squared	Root Mean Square Error (RMSE)
	ρ	*β*	*N*_∞_		
MR-BF	7.199	1.084	4.948 × 10^4^	0.996	0.039
MR-LF	7.565	1.005	4.948 × 10^4^	0.996	0.063
MR-PF	7.275	1.041	4.948 × 10^4^	0.996	0.068
MR-BPCF	5.407	1.382	5.000 × 10^4^	0.972	0.039
HMPR-BF	5.263	1.122	4.948 × 10^4^	0.996	0.030
HMPR-LF	7.518	1.182	4.948 × 10^4^	0.996	0.068
HMPR-PF	6.639	1.081	4.948 × 10^4^	0.996	0.041
HMPR-BPCF	5.263	1.119	4.948 × 10^4^	0.980	0.032
HIPR-BF	5.078	1.044	3.419 × 10^4^	0.977	0.049
HIPR-LF	9.394	0.655	4.948 × 10^4^	0.981	0.151
HIPR-PF	14.180	0.593	5.000 × 10^4^	0.998	0.277
HIPR-BPCF	16.131	1.133	5.000 × 10^4^	0.973	0.643

**Table 11:** Fitting parameters of the negative curvature phase.

Mixture Type	A	B	RMSE
MR-BF	0.303	0.283	0.015
MR-LF	0.429	0.238	0.027
MR-PF	0.570	0.199	0.054
MR-BPCF	0.616	0.187	0.021
HMPR-BF	0.486	0.203	0.062
HMPR-LF	0.691	0.196	0.052
HMPR-PF	0.585	0.190	0.047
HMPR-BPCF	0.488	0.204	0.070
HIPR-BF	0.507	0.182	0.030
HIPR-LF	0.423	0.182	0.056
HIPR-PF	0.41	0.193	0.074
HIPR-BPCF	0.928	0.248	0.066

**Table 12:** Line segments and feature points under the new method.

Mixture Type	First Line Segment	(PCP, RD)	Second Line Segment	(SIP, RD)
MR-BF	$y_{1}={5.44\times 10}^{-4}x$	(810, 1.95)	$y_{2}=3.09\times 10^{-4}x+1.71$	(14,500, 5.96)
MR-LF	$y_{1}=5.65\times 10^{-4}x$	(780, 1.40)	$y_{2}=3.42\times 10^{-4}x+1.50$	(14,325, 6.11)
MR-PF	$y_{1}=5.47\times 10^{-4}x$	(790, 1.43)	$y_{2}=3.22\times 10^{-4}x+1.57$	(14,410, 5.95)
MR-BPCF	$y_{1}=3.84\times 10^{-4}x$	(950, 1.63)	$y_{1}=2.11\times 10^{-4}x+1.56$	(18,120, 5.35)
HMPR-BF	$y_{1}=3.99\times 10^{-4}x$	(810, 1.16)	$y_{2}=2.21\times 10^{-4}x+1.33$	(14,615, 4.41)
HMPR-LF	$y_{1}=5.73\times 10^{-4}x$	(820, 1.81)	$y_{2}=3.03\times 10^{-4}x+2.11$	(14,780, 6.41)
HMPR-PF	$y_{1}=5.02\times 10^{-4}x$	(800, 1.39)	$y_{2}=2.86\times 10^{-4}x+1.56$	(14,515, 5.50)
HMPR-BPCF	$y_{1}=4.00\times 10^{-4}x$	(810, 1.16)	$y_{2}=2.21\times 10^{-4}x+1.33$	(14,610, 4.41)
HIPR-BF	$y_{1}=5.53\times 10^{-4}x$	(550, 1.00)	$y_{2}=4.32\times 10^{-4}x+0.59$	(13,960, 5.64)
HIPR-LF	$y_{1}=5.73\times 10^{-4}x$	(570, 0.63)	$y_{2}=5.40\times 10^{-4}x+0.14$	(13,260, 6.17)
HIPR-PF	$y_{1}=7.79\times 10^{-4}x$	(505, 0.63)	$y_{2}=8.27\times 10^{-4}x-0.16$	(12,930, 8.56)
HIPR-BPCF	$y_{1}=1.30\times 10^{-3}x$	(600, 4.34)	$y_{2}=6.82\times 10^{-4}x+4.11$	(14,530, 13.4)

**Table 13:** Price list of each maintenance process.

Raw Materials	Cost (¥/t)	Construction Technics	Cost (¥/t)
Asphalt	4500	Milling	25
Aggregate	250	Mixing	80
Regeneration agent	16,000	Transportation	5
BF	22,000	Vehicle group rental	160
LF	4200	Vehicle group fuel consumption	58
PF	5500	Paving and rolling	40

**Table 14:** Cost details of each maintenance process.

Mixture Type	Milling	Asphalt Mixture	Mixing	Paving and Rolling	Transportation
MR-BF	25	562.5	80	40	5
MR-LF	25	517.6	80	40	5
MR-PF	25	513.0	80	40	5
MR-BPCF	25	537.8	80	40	5
HMPR-BF	25	413.7	80	40	10
HMPR-LF	25	382.9	80	40	10
HMPR-PF	25	379.1	80	40	10
HMPR-BPCF	25	396.4	80	40	10
HIPR-BF	0	163.5	0	0	223
HIPR-LF	0	149.6	0	0	223
HIPR-PF	0	150.4	0	0	223
HIPR-BPCF	0	155.3	0	0	223

## Data Availability

Not applicable.

## Abbreviations

For reading convenience, a list of abbreviations is given below.

**Abbreviations**	**Full Form**
AFM	Atomic force microscopy
BF	basalt fiber
BPCF	Basalt-polyester compound fiber
CS	Creep stage
HIPR	Hot in-place recycling
HMA	Hot mix asphalt
HMPR	Hot mix plant recycling
HRAM	Hot rejuvenated asphalt mixtures
HWTT	Hamburg wheel tracking test
LF	Lignin fiber
LVDT	Linear variable differential displacement transducer
MR	Milling resurfacing
$N_{SN}$	Intersection point
PCP	Post-compaction phase
PCS	Post-compaction stage
PF	Polyester fiber
RAP	Reclaimed asphalt pavement
RD	Rutting depth
RMP	Regeneration maintenance processes
RTFOT	Rolling thin film oven test
SGC	Super-pave gyratory compactor
SIP	Stripping inflection point
SS	Stripping stage
TRD	Total rut depth
